# Supplementary material for: Characterization of Avian Influenza Viruses Detected in Kenyan Live Bird Markets and Wild Bird Habitats Reveal Genetically Diverse Subtypes and High Proportion of A(H9N2), 2018–2020
Source: Viruses. 2024 Sep 5;16(9):1417. doi: 10.3390/v16091417 (PMC11436075; doi:10.3390/v16091417)
Supplement: Supplementary file 1 [file viruses-16-01417-s001.zip › Predominant Species by Lake- 2020.pdf]

Table S1- List of predominant bird species per Lake (Feb/Mar 2020)

Name of Lake	Sampling Site Name	Predominant bird species (Scientific name)	Distribution of Predominant bird species
**Lake Naivasha**	Wetland	Egyptian Goose (*Alopochen aegyptiaca)*	Resident (Local)
	Round house	Egyptian Goose (*Alopochen aegyptiaca)*	Resident (Local)
	Banda	Egyptian Goose *(Alopochen aegyptiaca)*	Resident (Local)
	Crescent Island	Grey-headed Gull (*Chroicocephalus cirrocephalus)*	Palearctic
	Oloiden	Great Cormorant (*Phalacrocorax carbo)*	Palearctic
**Lake Elementaita**	Kikopey shore	Grey-headed Gull (*Chroicocephalus cirrocephalus)*	Palearctic
	Maji moto shore	Great White Pelican (*Pelecanus onocrotalus)*	Palearctic
	Serena shore	Little Stint (*Calidris minuta)* PM	Palearctic
**Lake Nakuru**	Nderit inlet	Great White Pelican (*Pelecanus onocrotalus)*	Palearctic
	WCK shore	Little Egret *(Egretta garzetta)*	Palearctic
	Makalia inlet	Yellow-billed Stork (*Mycteria ibis)*	Palearctic
	Njoro inlet	Great White Pelican (*Pelecanus onocrotalus)*	Palearctic
	Main Gate	Egyptian goose (*Alopochen aegyptiaca*)	Resident (Local)
**Lake Bogoria**	Main Gate	Lesser flamingo (*Phoeniconaias minor*)	Palearctic
	Hotsprings	Lesser flamingo (*Phoeniconaias minor*)	Palearctic
**Lake Victoria**	Bunyala	Long Toed Plover (*Vanellus crassirostris)*	Palearctic
	Siungu	White Winged black Terns (*Chlidonias leucopterus)*	Palearctic
	Bunyala 1	White Winged black Terns (*Chlidonias leucopterus)*	Palearctic
	Ahero	Glossy Ibis (*Plegadis falcinellus*)	Resident (Local)
	Ahero 2	Ruffs (*Calidris pugnax*)	Palearctic
